# Streamlining referrals by establishing a UAE-specific referral algorithm for CVD patients with overlapping COPD: a collaborative effort by cardiologists and pulmonologists

**DOI:** 10.3389/fcvm.2025.1531966

**Published:** 2025-06-24

**Authors:** Abdulla Shehab, Ashraf Alzaabi, Abdulmajeed Al Zubaidi, Bassam Mahboub, Hadi Skouri, Hamad Alhameli, Hassan El-Tamimi, Mohammed Nizam Iqbal

**Affiliations:** ^1^ Department of Cardiology, Burjeel Hospital, Al Ain, United Arab Emirates; ^2^ Department of Pulmonology, Zayed Military Hospital, Abu Dhabi, United Arab Emirates; ^3^ Department of Cardiology, Burjeel Hospital, Abu Dhabi, United Arab Emirates; ^4^ Department of Pulmonology, Rashid Hospital, Dubai, United Arab Emirates; ^5^ Department of Cardiology, Sheikh Shakhbout Medical City, Abu Dhabi, United Arab Emirates; ^6^ Department of Pulmonology, Cleveland Clinic, Abu Dhabi, United Arab Emirates; ^7^ Department of Cardiology, Mediclinic Hospital, Dubai, United Arab Emirates

**Keywords:** cardiovascular, COPD, United Arab Emirates, collaboration, cardiologists, pulmonologists

## Abstract

**Introduction:**

Concomitant COPD and CVD are highly prevalent and contribute to increased risk of hospitalizations, morbidity, and mortality, and impose a significant financial burden on healthcare systems. Diagnosis of COPD in patients with comorbid CVD and vice versa is challenging due to an overlap between the risk factors and symptoms of these two conditions. This 8-member task force comprising pulmonologists and cardiologists agreed that in the UAE, while COPD patients suspected of having CVD are promptly referred to cardiology, CVD patients who may potentially also have COPD are often not referred or referred late from cardiology to pulmonology. This gap in identifying CVD patients who may potentially also have COPD must be addressed to facilitate referrals of such patients to pulmonology.

**Methods and results:**

A task force comprising an equal number of cardiologists and pulmonologists met virtually and identified the gaps in current practices for diagnosing patients with comorbid COPD and CVD in the UAE. The task force has proposed an algorithm to expedite the referral of CVD patients suspected of COPD from cardiology to pulmonology.

**Conclusion:**

Implementing this referral algorithm across all cardiology departments in the UAE can facilitate the diagnosis of COPD in CVD patients, allow timely treatment of COPD, and improve patient outcomes.

## 1 Introduction

### 1.1 Global burden of COPD

Chronic obstructive pulmonary disease (COPD) is a progressive respiratory condition that causes airflow limitation due to abnormalities in the airways and/or alveoli. This results in persistent respiratory symptoms, including dyspnea, cough, and sputum production, often leading to acute episodes of worsening symptoms or exacerbations (1). The burden associated with COPD is multifaceted as it significantly affects the quality of life of patients, adversely impacts their caregivers/families, and imposes financial strain on healthcare systems. According to the World Health Organization (WHO) estimates, COPD was responsible for 3.23 million deaths globally in 2019, making it the third leading cause of death.Click or tap here to enter text (2). The global prevalence of COPD among individuals between the ages of 30 and 79 has been estimated to be around 7.6% according to the lower limit of normal (LLN) definition (FEV_1_/FVC < LLN; FEV1-forced expiratory volume in one second; FVC-forced vital capacity) and 10.3% based on the Global Initiative for Chronic Obstructive Lung Disease (GOLD) case definition (FEV_1_/FVC < 0.7). These statistics translate to nearly 290 to 390 million people affected by COPD globally, irrespective of the definition used to diagnose COPD (3).

• Acute exacerbations associated with COPD can lead to hospitalization, which in turn increases the risk of hospital readmission in the future. Studies from the UK and US show that almost a quarter of COPD patients hospitalized for an acute exacerbation require hospital readmission within 30 days of discharge. About one-third of those hospitalized for an acute exacerbation are readmitted within 90 days from the initial episode. Moreover, in the UK, the 90-day readmission rate has jumped substantially from 33% in 2008 to 43% in 2014 (4).

The need for hospitalization and increased risk of readmission impose a substantial economic burden on healthcare systems and patients. A study aiming to assess the financial impact of COPD, considering the expected prevalence based on current estimates, revealed that over 30 years starting from 2020, COPD would cost the world economy INT$4.326 trillion (5). The potential increase in the economic burden associated with COPD over the next three decades underscores the importance of prioritizing resources towards effective interventions, including preventive care, early detection, prompt referrals, and optimal treatment strategies. Investing in these interventions now will have positive economic and public health implications in the future.

### 1.2 Disease burden associated with COPD: UAE perspective

Although the prevalence data from the UAE are limited, they provide a broad overview of the disease burden and financial impact of COPD. The prevalence of COPD in the MENA (Middle East and North Africa) region is estimated to be about 6%, varying between 1.9% in the UAE and 6.1% in Syria (6). Other studies have found the COPD prevalence in the UAE to vary between 3.7% and 5.3% (7). Also, the impact of COPD on healthcare utilization and costs in the UAE is substantial. Hospitalizations, emergency department, outpatient and general practitioner visits, and prescription drug expenses collectively contribute towards the direct medical costs associated with COPD. Finkelstein et al. have estimated the annual total direct medical costs of COPD and 6 other noncommunicable diseases (coronary heart disease, stroke, type 2 diabetes mellitus, breast cancer, colon cancer, and asthma) in the UAE to be $4.371 billion. Of these direct costs, 13.2% can be solely attributed to COPD, based on an estimated prevalence of 2.1%. On the other hand, coronary heart disease, with an estimated prevalence of 2.2%, accounts for only 6.9% of the cost. Apart from the direct medical costs, COPD-related indirect costs due to productivity losses as a result of lost work days and reduced output at work amount to a staggering $3.942 billion (see Table 1) (8).

**Table 1:** Estimated direct medical costs and indirect costs due to annual productivity losses associated with COPD in UAE.

Cost category	Estimated value (USD)
Total estimated direct medical costs due to the 7 NCDs	$4.371 billion
COPD-related total estimated direct medical costs	$577 million
Total estimated annual productivity losses due to the 7 NCDs	$22.1 billion
COPD-related estimated annual productivity losses	$3.942 billion
• COPD-related estimated indirect costs due to lost days at work	$1.032 billion
• COPD-related estimated indirect costs due to reduced output at work	$2.910 billion

NCDs, noncommunicable diseases (coronary heart disease, stroke, type 2 diabetes mellitus, breast cancer, colon cancer, COPD and asthma); COPD, chronic obstructive pulmonary disease.

Adapted with permission from Finkelstein et al. (8)

The financial impact (direct and indirect costs) of nearly $4.5 billion highlights the burden COPD imposes on the UAE economy and the public health challenge it poses. Efforts must be taken to identify areas of improvement in COPD diagnosis and management to enhance patient outcomes while reducing the economic impact of COPD.

### 1.3 Risk factors for COPD

Several risk factors that contribute to the development and progression of COPD have been identified and are listed in Table 2 (9–11). While smoking is widely recognized as the primary risk factor for the development of COPD, approximately 25%–45% of COPD patients are nonsmokers, highlighting the role of other factors such as environmental exposures, occupational hazards, and a history of respiratory infections (9). Also, variations in exposure to these risk factors among populations from different geographical regions lead to disparities in COPD outcomes (12). The need to reduce health disparities in COPD by minimizing the exposure to modifiable individual and environmental risk factors contributing to poor outcomes cannot be emphasized enough.

**Table 2:** Risk factors associated with the development and progression of COPD.

• Smoking
• Exposure to second-hand smoke/passive smoking
• Pollution from ambient particulate matter
• Occupational exposure to vapors/particulate matter/gases/fumes/dust
• Air pollution from solid fuels/biomass
• Ambient ozone pollution
• Temperature extremes
• Previous diagnosis of asthma or tuberculosis
• History of hospitalizations due to childhood respiratory infections
• High temperature

COPD, chronic obstructive pulmonary disease.

Murray et al. (10).

Safiri et al. (11).

Pando-Sandoval et al. (9).

Konstantinoudis et al. (13).

### 1.4 Risk factors specific to the GCC-MENA region

Air pollution, exposure to dust and fumes, biomass-smoke inhalation, smoking, second-hand smoke, and previous tuberculosis infection are important factors associated with increased COPD risk in the GCC–MENA region (6). Studies also suggest a link between heat exposure and COPD hospitalizations, which is potentially relevant in this region. A study in England showed that for every 1°C increase above a summer temperature of 23.2°C, the risk of COPD-related hospitalization increased by 1.47% (13). The fast-paced urbanization and development in the GCC-MENA region have led to increased vehicular traffic and rapid construction activities, causing a rise in outdoor pollution. Although no longer a major concern in urban areas, biomass smoke may be a crucial risk factor for COPD in the elderly, who may have used biomass fuels in the past. Dust storms in the UAE may also contribute to acute COPD exacerbations (6).

Apart from environmental factors, genetic factors play a crucial role in COPD development and progression. Despite the large number of expatriates in the UAE population, the specific genetic factors contributing to COPD development in this diverse patient population remain unknown. Genetic and epidemiological studies addressing this knowledge gap can provide insights into the interplay between genetics, environment, and COPD risk in the UAE population and can be undertaken in the future (6).

COPD imposes a substantial socioeconomic burden on patients and healthcare systems, with multiple risk factors apart from smoking contributing to its development and progression. Also, it is noteworthy that mortality associated with COPD is primarily attributable to cardiovascular complications rather than respiratory failure (14).

### 1.5 Prevalence of COPD and CVD as comorbidities

**Figure 1:**
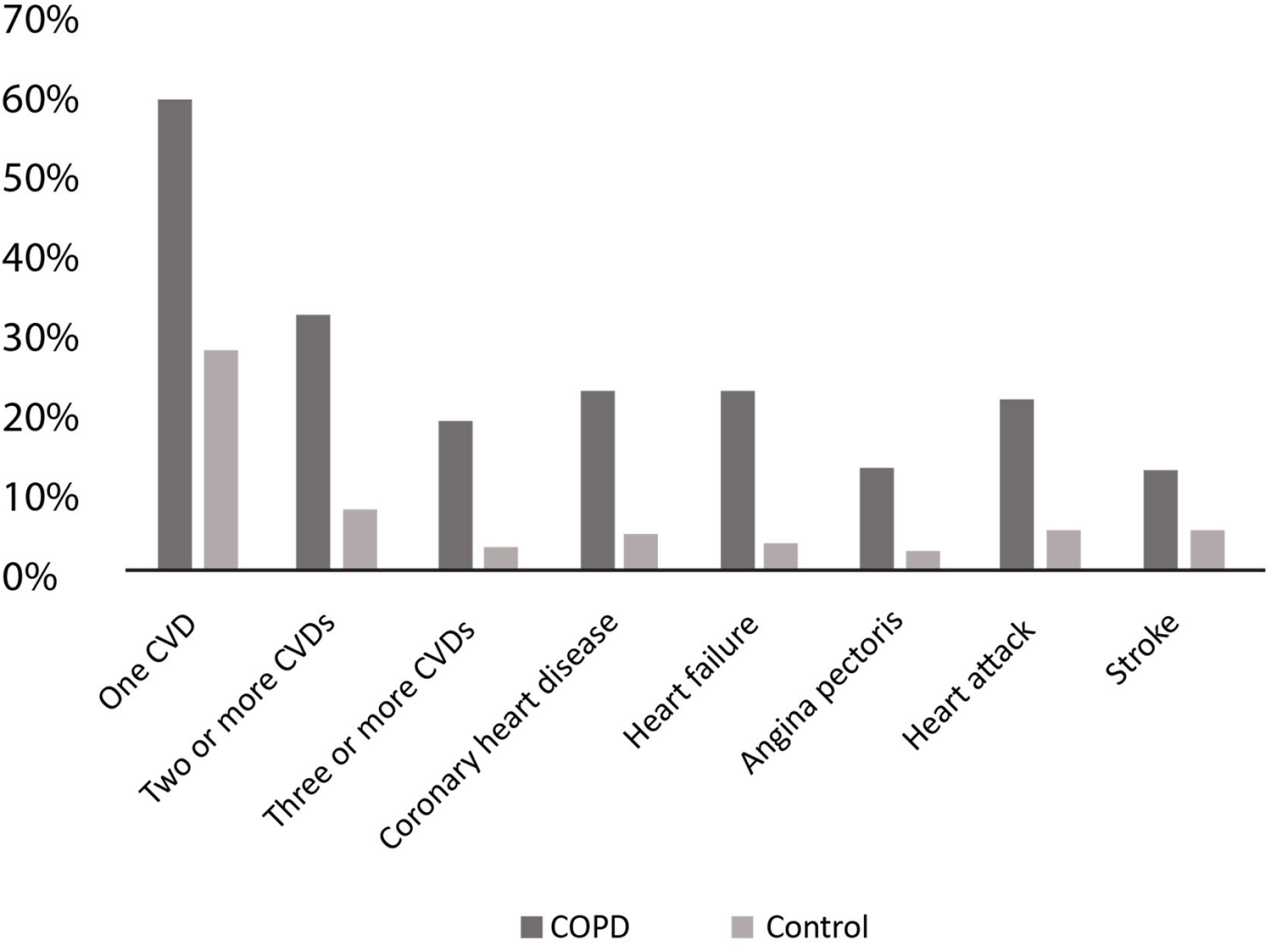
Prevalence of different CVDs in COPD patients. CVD, cardiovascular disease; COPD, chronic obstructive pulmonary disease. Adapted with permission from: Chen et al. (15).

The high prevalence of COPD and cardiovascular disease (CVD) as multimorbidities exacerbates the clinical burden and complicates management strategies. Studies have shown that compared to individuals without COPD, COPD patients have a higher prevalence of one or more CVDs (see Figure 1) (15).

The BREATHE study, a large cross-sectional, observational, population-based survey of COPD, was conducted in 2012 across 11 countries (10 in the MENA region and Pakistan) (16). An analysis of the study participants by Mahboub et al. revealed that COPD patients were more likely to report comorbidities compared to those without COPD (55.2% vs. 39.1%, *p* < 0.0001). Moreover, CVD was the most commonly reported comorbidity in this cohort, with a prevalence of 28.8% among female participants and 18.3% among male participants (17).

Likewise, several studies have also established the high prevalence of COPD in people with CVD (18–22). The prevalence of COPD has been shown to range between 13% and 39% in patients with heart failure (HF) (18, 19). A study by Bektas et al. showed that among patients with comparable HF severities, those with comorbid COPD exhibited more dyspnea and had poorer quality of life compared to HF patients without COPD. Moreover, among those identified as having comorbid COPD, 63% were previously undiagnosed (20). COPD is also highly prevalent in those with established ischemic heart disease (IHD), with a prevalence ranging from 6.5% to 30.5% (18, 19). In patients diagnosed with atrial fibrillation (AF), most studies estimate the COPD prevalence to be between 10% and 15%; some studies suggest it could even be >20%, especially in patients over 65 years (18, 19). The coexistence of AF and COPD has also been associated with additional comorbidities, primarily diabetes mellitus and chronic heart failure (21). Additionally, COPD contributes to poor short-term outcomes, increased risk of hospitalization, stroke, and all-cause mortality in AF patients (22).

### 1.6 Pathophysiology of the COPD-CVD link

The concomitant presence of COPD and CVD is highly prevalent and is associated with significant morbidity and mortality (23). The presence of CVD has been shown to adversely affect COPD outcomes and vice-versa (14). The pathophysiological processes linking COPD and CVD identified so far are complex, as they involve several shared pathways that uniquely affect the development and progression of each condition while simultaneously having a tangential impact on the other. Studies now show that the syndemic link between these two chronic conditions goes beyond shared risk factors like smoking and involves more complex interactions between the cardiac and respiratory systems, with systemic inflammation being a crucial component (23, 24). Systemic inflammation as a result of noxious stimuli leads to atherosclerosis and chronic bronchitis, triggering acute cardiac and respiratory events that can cause structural remodeling and, consequently, heart or respiratory failure (23).

An interplay between several other pathophysiological processes set in motion by COPD leads to lung hyperinflation, vascular endothelial injury, arterial stiffness, and chronic or intermittent hypoxia, which directly or indirectly affect cardiovascular functioning. Thus, given the intricate association between the COPD and CVD pathophysiological mechanisms, COPD patients are at a higher risk of cardiac disease than patients with no COPD (14, 23).

### 1.7 COPD, CVD, and mortality

**Figure 2:**
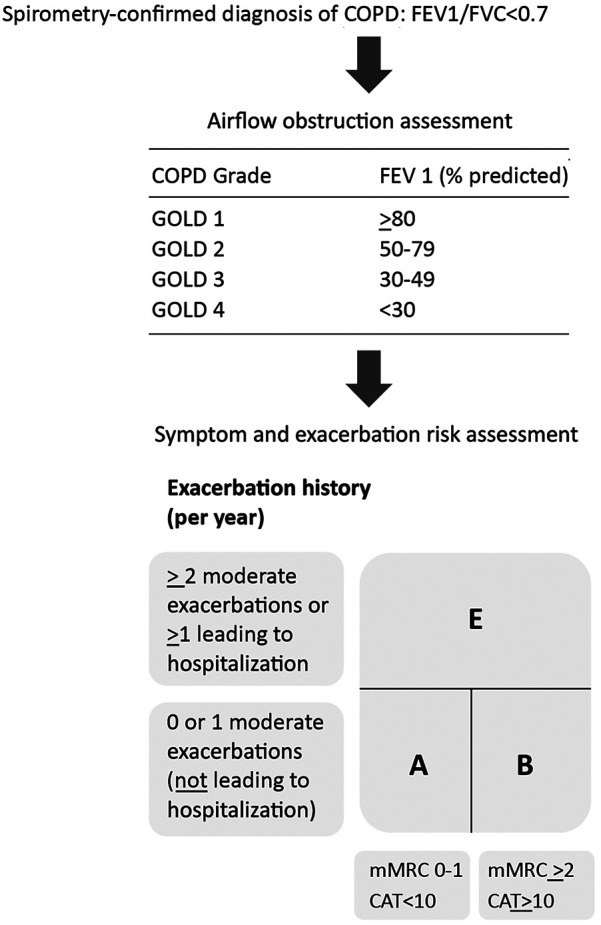
GOLD 2024-proposed COPD assessment tool. FEV1, forced expiratory volume in 1 s; FVC, forced vital capacity; mMRC, modified medical research council dyspnea scale (a questionnaire to measure breathlessness); CAT™: COPD assessment Test (8-item questionnaire to assess the health status of COPD patients). Adapted from: Global Initiative for Chronic Obstructive Lung Disease (1). http://goldcopd.org/2024-gold-report/ ©2023, 2024, Global Initiative for Chronic Obstructive Lung Disease, available from https://www.goldcopd.org, published in Deer Park, IL, USA.

A diagnosis of COPD is suspected in patients with dyspnea, chronic cough/sputum production, recurrent lower respiratory tract infections, and the presence of COPD risk factors. However, COPD diagnosis is confirmed by spirometry (post-bronchodilator FEV1/FVC < 0.7). Stratification of COPD patients into GOLD stages 1 to 4 is based on the extent of airflow obstruction, assessed by decreasing FEV1 as a percentage of the predicted value. Additionally, the GOLD-proposed COPD combined assessment tool integrates exacerbation history and symptom level while evaluating disease severity and is shown in Figure 2 (1).

Acute exacerbations requiring hospitalization are indicative of worsening COPD symptoms and have been associated with an increased risk of CVD events as well as mortality (25, 26). Kunisaki et al. have shown that the risk for CVD events increased in the first 30 days after an acute exacerbation. Moreover, the risk during the first month was more than double in patients hospitalized for their exacerbation compared to those who were not. The risk for CVD events remained elevated for one year after an acute exacerbation in both patient groups—those who required hospitalization for their acute exacerbations and those who did not (25).

A study by Szylinska et al. evaluating the incidence of complications and mortality in patients with acute ischemic stroke (AIS) found that the risk of cardiac and pulmonary complications after a stroke was significantly higher in COPD patients. Also, their one-year survival probability was significantly lower compared to those with no COPD (27). Yoshihisa et al. compared the mortality outcomes of hospitalized HF patients who were diagnosed with spirometry as having mild COPD (GOLD stage (1), moderate COPD (GOLD stage (2), or no COPD. They identified moderate COPD, based on the GOLD stage 2 definition, as an independent predictor of cardiac death, non-cardiac death, and all-cause mortality in patients with HF (28).

Further evaluation of the relationship between acute exacerbations of COPD (AECOPD) and mortality by Rothnie et al. revealed that both frequency and severity of AECOPDs at baseline were predictive of future exacerbation rate and mortality. Analysis of a 12-month period before death in case-control subjects revealed that ≥2 moderate exacerbations increased the risk of death, and this risk was doubled in those experiencing ≥5 moderate AECOPDs. Moreover, the risk of death was 14 times higher in those who had a severe AECOPD in the previous 12 months compared with those who had no AECOPD. It was also observed that nearly one-quarter of the patients in this study did not experience an exacerbation during the follow-up period, implying that patients responding well to treatment may potentially remain exacerbation-free (29).

The findings of Rothnie et al. were also confirmed by another large observational COPD cohort study by Whittaker et al. using the data from electronic healthcare registries (EHR) from the UK. This study also showed that the greater the number and severity of COPD exacerbations at baseline, the higher the risk of future exacerbations, all-cause mortality, and COPD-related mortality. A trend towards increased CVD mortality was also seen with an increase in the frequency of moderate exacerbations, but only in those experiencing up to 2 severe exacerbations. On the other hand, patients with ≥3 severe exacerbations at baseline were more likely to have COPD listed as the cause of death on their death certificate (26).

Another large cohort study from England comprising nearly 32,000 COPD patients demonstrated an independent association between COPD and IHD, HF, atrial fibrillation, and peripheral vascular disease. Additionally, these cardiovascular conditions were independently associated with all-cause mortality in COPD patients (30). Symptomatic COPD patients with concomitant CVD may thus be at a higher risk of adverse outcomes and mortality.

All these findings underscore the importance of early diagnosis and management of COPD patients with concomitant CVD who may seek care from cardiologists but potentially remain undiagnosed for COPD and, thus, at risk for COPD disease progression and CVD-related mortality.

### 1.8 Challenges in diagnosing COPD—overshadowed by CVD?

Limited data on the prevalence, incidence, and mortality of COPD in the Gulf region suggest that it is likely underdiagnosed (31). In the UAE, this has been attributed to a combination of factors such as the absence of standardized diagnostic protocols, low level of awareness about the condition, indifferent attitude toward early symptoms, high rates of smoking, environmental pollution, and misdiagnosis of COPD as asthma (32).

In a Canadian study that included 895,155 individuals with physician-diagnosed COPD, 6.3% did not receive any ambulatory care, and 89.6%, 10.7%, 24.5%, and 82.3% sought care from primary care physicians, pulmonologists, cardiologists, and other specialists, respectively. As opposed to 43.7% of patients requiring two or more hospitalizations, only 9.9% of those not requiring hospitalization approached pulmonologists for specialist care (33). These statistics reveal that COPD patients are less likely to receive appropriate specialist care in the early stages of the disease, often seeking specialist intervention only when their condition is severe enough to warrant hospitalization. The findings of this study also suggest that COPD patients would be more inclined to consult cardiologists rather than pulmonologists, and this trend may be even more pronounced in patients with comorbid CVD.

While several guidelines provide recommendations for the differential diagnosis of COPD and CVD, algorithms guiding the diagnosis of COPD in patients with comorbid CVD, or vice-versa, are not available (34). This gap poses a significant challenge for healthcare providers in accurately identifying one comorbidity in the presence of the other, potentially leading to underdiagnosis, sub-optimal management, poor outcomes, and increased mortality risk. COPD patients and physicians may often attribute symptoms, such as dyspnea, solely to the underlying cardiac condition, thereby allowing COPD to remain undiagnosed. The symptoms of CVD can thus mask the presence of COPD, leaving it undetected (35).

### 1.9 Management of COPD: a quick overview

**Figure 3:**
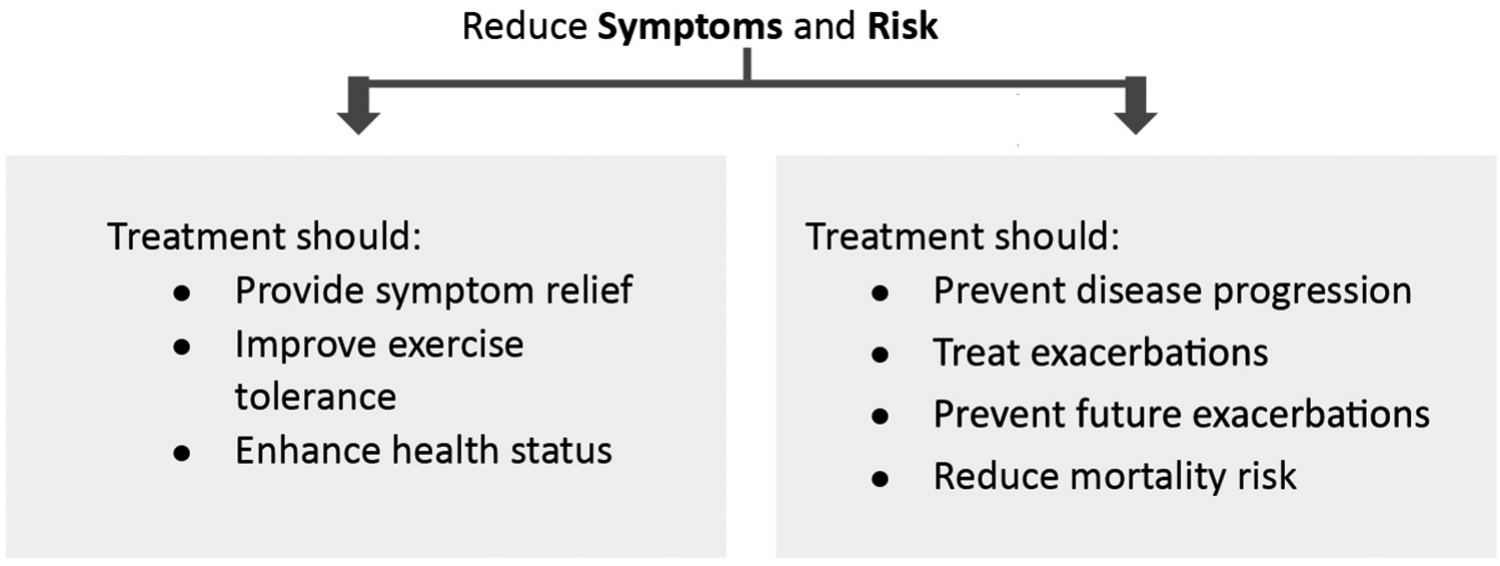
Stable COPD treatment goals. Adapted with permission from: Global initiative for Chronic Obstructive Lung Disease (1). http://goldcopd.org/2024-gold-report/ ©2023, 2024, Global Initiative for Chronic Obstructive Lung Disease, available from https://www.goldcopd.org, published in Deer Park, IL, USA.

Effective management of COPD entails making an accurate diagnosis, taking a comprehensive patient history, and selecting the most appropriate treatment option after evaluating its benefits, risks, availability, and cost. Treatment success also depends on educating patients about COPD, emphasizing the importance of treatment compliance, and reducing exposure to risk factors. The 2024 GOLD report highlights the importance of tailoring COPD treatment strategies to patients' severity of symptoms and history of exacerbations. Primary treatment goals should focus on mitigating symptoms and minimizing the risk of future exacerbations (Figure 3) (1).

**Figure 4:**
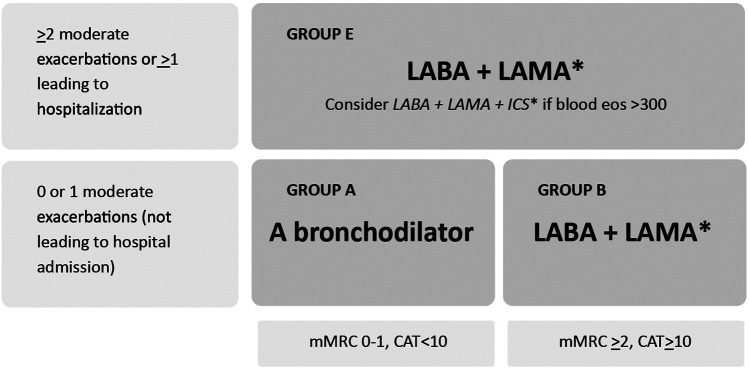
Initial pharmacological treatment based on GOLD group. Due to improved treatment adherence, single inhalers may be more effective and convenient to patients than multiple inhalers. Exacerbations: Number of Exacerbations/year. Eos: Blood eosinophil count (cells/ul); mMRC: Modified Medical Research Council dyspnea questionnaire; CAT™: COPD Assessment test™. Adapted from: Global Initiative for Chronic Obstructive Lung Disease (1). http://goldcopd.org/2024-gold-report/ ©2023, 2024, Global Initiative for Chronic Obstructive Lung Disease, available from https://www.goldcopd.org, published in Deer Park, IL, USA.

Treatment must be tailored according to the GOLD group a patient is categorized into based on exacerbation risk and symptom severity (1). Initial pharmacological treatment for GOLD groups A, B, and E is shown in Figure 4.

Additionally, two fixed-dose inhaled combination triple therapies —budesonide/glycopyrrolate/formoterol fumarate (BGF) and fluticasone furoate/umeclidinium/vilanterol (FF/UMEC/VI) — have been shown to reduce all-cause mortality compared to dual therapy (36, 37). Based on the evidence from the ETHOS and IMPACT trials, the GOLD 2024 report recommends considering the use of these therapies over dual bronchodilation therapy in COPD patients categorized as Group E (Figure 4) and having a blood eosinophil count ≥ 300 cells/µl (Table 3) (1, 36, 37).

**Table 3:** GOLD 2024: evidence supporting mortality reduction in COPD patients.

Therapy	RCT	Treatment effect on mortality	Patient characteristics
LABA + LAMA + ICS	Yes	Single inhaler triple therapy vs. dual inhaled long-acting bronchodilation therapy; relative risk reduction IMPACT: HR 0.72 (95% CI: 0.53–0.99; *p* = 0.042) ETHOS: HR 0.51 (95% CI:0.33–0.80; unadjusted *p* = 0.0035)	Symptomatic people with a history of frequent and/or severe exacerbations

LABA, long acting β2 agonist; LAMA, long-acting muscarinic antagonist; ICS, inhaled corticosteroid; HR, hazard ratio; CI, confidence interval.

Adapted with permission from: Global initiative for Chronic Obstructive Lung Disease (1).

©2023, 2024, Global Initiative for Chronic Obstructive Lung Disease, available from https://www.goldcopd.org, published in Deer Park, IL, USA.

Lipson et al. (36).

Martinez et al. (37).

GOLD 2024 practical recommendation: Consider LABA + LAMA + ICS in Group E COPD patients if blood eosinophil count >300 cells/ul.

### 1.10 The vital role of cardiologists in enhancing early diagnosis of COPD

Given the high prevalence of COPD in CVD patients, many patients seeking care in cardiology may also have underlying COPD that must be diagnosed and managed. Optimal management of COPD is crucial as it can also improve cardiovascular outcomes. Conversely, sub-optimally treated or untreated COPD can worsen outcomes. Smaller studies conducted to objectively assess lung function in HF patients have found that nearly half of them had abnormal spirometry results (35). This finding emphasizes the significant role cardiologists must play in assessing COPD in patients presenting with CVD and promptly referring them to pulmonologists for further evaluation and management (38). COPD is a significant yet frequently overlooked comorbidity in CVD patients. Despite being highly prevalent and manageable, COPD often remains undiagnosed, resulting in poor patient outcomes. It is crucial to take proactive steps to ensure COPD does not evade diagnosis in CVD patients and that suspected patients are promptly referred to pulmonologists to ensure optimal and timely management.

This paper aims to establish a UAE-specific consensus referral algorithm with the help of an expert panel comprising cardiologists and pulmonologists. The availability of such an algorithm can make referring patients from cardiology to pulmonology in the UAE seamless.

## 2 Methods

An 8-member task force comprising an equal number of cardiologists and pulmonologists from the UAE met virtually on March 27, 2024, to discuss data on poor outcomes in patients with overlapping COPD and CVD, review the recent updates in the management of COPD based on published evidence, and identify the gaps in current practices for diagnosing patients with comorbid COPD and CVD in the UAE. The cardiologists’ and pulmonologists' perspectives were discussed to comprehensively understand the challenges encountered in diagnosing CVD patients with comorbid COPD and vice-versa. Key discussion points included the potential impact of untreated COPD on patients' quality of life and cardiopulmonary risk, the diagnostic challenges in patients with overlapping COPD and CVD, and the role of a multidisciplinary approach in identifying and managing these patients to improve outcomes. This discussion highlighted critical gaps in the current clinical practice in the UAE, especially the need for increased suspicion and awareness regarding the potential overlap of COPD in CVD patients.

The panelists agreed that developing a national consensus referral algorithm, which can be implemented across cardiology departments in the UAE, would be the most optimal strategy to enhance COPD diagnosis in patients with CVD. All panelists contributed equally to the development of the referral algorithm presented in this manuscript.

## 3 Results

**Figure 5:**
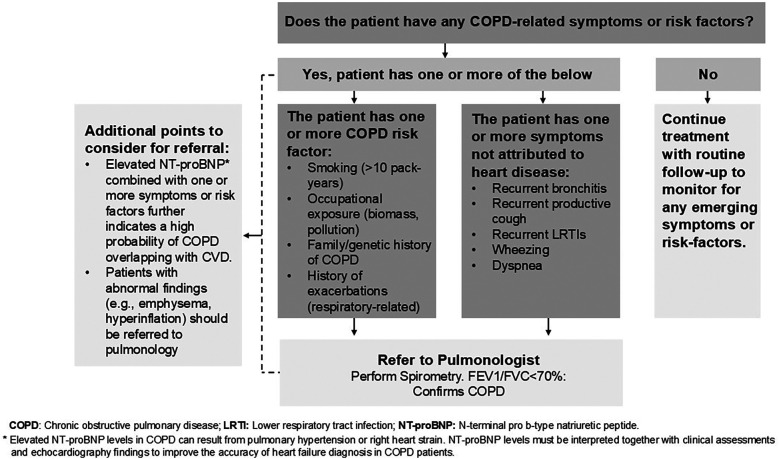
Referral algorithm for stable CVD patients with suspected COPD overlap.

All the pulmonologists and cardiologists on this panel agreed that the threshold for referral from pulmonology to cardiology in the UAE is low, which means that pulmonologists readily refer COPD patients suspected of having CVD to cardiologists for assessment, even if the suspicion is not very strong. On the other hand, CVD symptoms overshadow COPD symptoms, and cardiologists in the UAE often do not suspect COPD in CVD patients. Thus, the panel believes that the referral of CVD patients with possible comorbid COPD from cardiology to pulmonology needs further optimization. Considering the context of the UAE healthcare system, the panel developed and unanimously agreed on the referral algorithm depicted in Figure 5. By facilitating patient referrals from cardiology to pulmonology, this algorithm can enhance the diagnosis and management of COPD, ultimately leading to improved long-term outcomes.

While prompt referrals from cardiology to pulmonology are crucial and need optimization in the UAE healthcare context, the panel also acknowledges the importance of timely referrals from pulmonology to cardiology when necessary. The panel recommends cardiovascular risk assessments in COPD patients, especially those with risk factors or reduced exercise tolerance. COPD patients presenting with chest pain, disproportionate dyspnea, or palpitations must be suspected of having underlying CVD (39). Electrocardiographic (ECG) screening, exercise stress testing, and myocardial perfusion imaging can be non-invasive options considered for detecting CVD, including silent ischemia. Timely referrals to cardiology are also crucial in COPD patients with overlapping sleep disorders, such as obstructive sleep apnea (OSA), which is independently associated with CVD (40). Incidental findings in COPD patients, such as coronary artery calcifications detected during annual low-dose CT (LDCT) scans for lung cancer screening in individuals with a smoking history of ≥20 pack-years, warrant a prompt referral to cardiology for further assessment of CVD risk (1, 41).

Clinical presentation and abnormal lab findings should guide the decision to refer COPD patients to cardiology for further evaluation and management (39). Additionally, the panel emphasizes regular ECG screenings in patients receiving theophylline therapy and during acute exacerbations. Prompt referral to cardiology is essential when ECG findings are abnormal (1, 39).

## 4 Discussion

COPD puts an enormous economic burden on the UAE's healthcare system, both in terms of direct medical costs and indirect costs due to low productivity (8). Studies show that increasing COPD severity leads to more frequent hospitalizations, with comorbid CVD further driving longer hospital stays, more frequent ICU admissions, higher annual exacerbation-related costs, and greater costs per hospitalized exacerbation (42). A combination of the high prevalence of COPD and the significant disease burden associated with it poses a considerable public health challenge that must be addressed by identifying effective prevention strategies, improving healthcare access and referral processes, and implementing optimal management approaches.

Suboptimal awareness about COPD among the local population is another significant barrier to timely diagnosis. COPD awareness may have improved in the UAE since the results from the BREATHE study were published in 2012, which revealed that only a little over half of the analyzed participants from the MENA region were adequately aware of their respiratory condition (43). However, public awareness about COPD is still likely to be suboptimal (6, 44). Healthcare policies to promote public educational campaigns and community outreach programs are needed to enable individuals to recognize symptoms and seek prompt medical care.

The United States Preventive Services Task Force (USPSTF) recommends against routine COPD screening in asymptomatic individuals who do not recognize or report any respiratory symptoms, citing a lack of net benefit (45). However, this recommendation must be balanced against the potential risk of missed or delayed diagnosis, especially in high-risk populations where symptoms may overlap with other conditions like CVD. Although universal screening may not be practical, targeted screening approaches may offer a cost-effective solution in high-risk groups. The feasibility of implementing such strategies within the UAE healthcare system must be evaluated.

CVD and COPD share multiple risk factors, including smoking, and their pathophysiological processes intersect, adversely impacting each other's outcomes (14, 46). Although the prevalence of CVD and COPD as comorbidities is high, shared risk factors and pathophysiologies imply significant diagnostic challenges (38). Despite the availability of reliable tools to accurately diagnose COPD, its underdiagnosis, when present concomitantly with CVD, highlights the need for increased vigilance and monitoring in identifying CVD patients who may be candidates for COPD screening (6). Moreover, the 2023 GOLD update for practicing cardiologists highlights this significant gap in the diagnosis of COPD among CVD patients. It provides guidance for cardiologists on the diagnosis, initial management, and integration of COPD care within their practice, including when to refer patients for specialized pulmonary consultation (38). Additionally, the growing use of CT angiography to diagnose IHD has led to an increase in the detection of incidental extracardiac findings, primarily in the lungs. Emphysema, one of the common findings, presents a valuable opportunity for prompt referral from cardiology to pulmonology and can facilitate early evaluation and management of underlying COPD (47). It is also crucial to highlight that overlapping symptoms between COPD and CVD can limit the accuracy of COPD symptom assessment tools, such as the 8-item questionnaire, COPD assessment test (CAT™), and the dyspnea questionnaire, modified Medical Research Council (mMRC) dyspnea scale (1). While valuable in routine COPD assessment, these tests may not accurately reflect the symptom burden in patients with comorbid COPD-CVD, where dyspnea can originate from multiple sources. While this article focuses primarily on referral optimization, future work should explore the validation of multidimensional tools or composite indices that more effectively differentiate between respiratory and cardiac symptoms in this patient cohort.

In the UAE, the threshold of referring COPD patients with suspected CVD from pulmonology to cardiology is low and not a significant concern. On the other hand, the cardiologists and pulmonologists on the panel identified delayed referrals from cardiology to pulmonology in the UAE healthcare system as a significant barrier to optimal and timely management of patients with CVD who may have underlying COPD. These delays, or sometimes even lack of referrals, lead to late or missed COPD diagnoses and increase the risk of exacerbations, hospitalizations, and death. Given the availability of effective treatment options for COPD that can potentially prevent or reduce the number of exacerbations, improve patients' quality of life, and reduce their risk of hospitalization and death, timely referral is crucial (48). Prompt evaluation of patients with suspected COPD by a pulmonologist can promote early intervention and improve long-term outcomes. The panel has identified a simple approach to achieve this objective. Developed collaboratively by cardiologists and pulmonologists, the referral algorithm presented in this paper can assist cardiologists in identifying and referring patients with suspected COPD to pulmonology for diagnosis confirmation and treatment.

Although accurate CVD risk assessment in COPD patients is vital for optimal clinical management, it remains a critical gap, as some risk prediction tools, such as QRISK3, are designed for the general population and tend to underestimate CVD risk in the COPD patient cohort (46, 49). Given the increased risk, there is an urgent need to validate currently available CVD risk assessment tools in COPD patients without established CVD. Additionally, developing an integrated CVD risk assessment algorithm tailored to COPD patients that incorporates cardiovascular risk factors, exacerbation frequency, lung function, biomarkers, ECG, and chest imaging can facilitate risk stratification. Moreover, incidental findings from LDCT and CT-angiography offer valuable opportunities for risk assessment and referral to the appropriate specialty for further evaluation and should not be missed (46). Some studies investigating the relationship between blood eosinophil levels and CVD risk have found an increased CVD risk associated with elevated eosinophil counts in COPD patients. On the other hand, some studies report no independent association between elevated eosinophil counts and the presence or severity of CAD (50). Thus, this remains an area of ongoing research, and additional studies on the clinical implications of these findings are warranted.

Future studies assessing the impact of implementing this referral algorithm across cardiology departments in the UAE on clinical outcomes could help validate its efficacy. Furthermore, the evidence from these studies may pave the way for broader implementation of this referral algorithm in regions with similar referral challenges and healthcare contexts. The proposed referral algorithm reflects a collaborative effort between cardiologists and pulmonologists. Increased collaboration between cardiologists and pulmonologists practicing within healthcare institutions must be encouraged, and institutional and administrative support must be provided to facilitate the establishment of multidisciplinary COPD-cardiology clinics. Such clinics can help optimize care for patients with overlapping cardiovascular and respiratory conditions by integrating patients' medical records and facilitating joint consultations and case study discussions by specialists.

It is also essential to acknowledge the role of primary care physicians in identifying the early signs and symptoms of COPD. The use of simple tools such as the CAT™ and spirometry in primary care can support the early identification of patients at risk (1). Establishing clear referral criteria based on persistent respiratory symptoms, frequent exacerbations, or the presence of cardiovascular comorbidities can help streamline referrals from primary care to pulmonology.

## 5 Conclusion

Implementing this cardiologist-approved referral algorithm can help streamline the diagnosis of COPD in patients with comorbid CVD and enable timely treatment of COPD.

Apart from facilitating the implementation of this algorithm, policymakers in the UAE can allocate resources for training healthcare providers, establishing integrated care pathways, improving access to specialist care, and enhancing COPD awareness in the general UAE population. Collectively, these steps will be expected not only to lead to better patient outcomes but also to reduce healthcare costs.

Additionally, leveraging the available digital health tools such as electronic health records, telemedicine platforms, and artificial intelligence technologies to implement this referral algorithm in the UAE healthcare systems is the need of the hour to ensure optimized COPD management and resource utilization.

## Data Availability

The original contributions presented in the study are included in the article/Supplementary Material, further inquiries can be directed to the corresponding author.
